# Evaluation of unintentional injuries sustained by children: A hospital based study from Ankara-Turkey

**DOI:** 10.12669/pjms.293.3150

**Published:** 2013

**Authors:** Piyal Birgul, Mine Esin Ocaktan, Recep Akdur, Yilmaz Mustafa Soner, Ikinci Sevil, Celik Safa

**Affiliations:** 1 Piyal Birgul, MD, PhD, Associate Professor, Department of Public Health, Ankara University Faculty of Medicine, Ankara, Turkey.; 2 Mine Esin Ocaktan, MD, PhD, Associate Professor, Department of Public Health, Ankara University Faculty of Medicine, Ankara, Turkey.; 3 Recep Akdur, MD, Professor, Department of Public Health, Ankara University Faculty of Medicine, Ankara, Turkey.; 4 Yilmaz Mustafa Soner, MD, Research Assistant, Department of Public Health, Ankara University Faculty of Medicine, Ankara, Turkey.; 5 Ikinci Sevil, MD, Research Assistant, Department of Public Health, Ankara University Faculty of Medicine, Ankara, Turkey.; 6 Celik Safa, MD, Forensic Medicine Specialist, Council of Forensic Medicine Yenibosna, Istanbul, Turkey.

**Keywords:** Childhood unintentional injuries, Injury surveillance, Childhood injuries

## Abstract

***Objectives: ***Unintentional injuries are one of the leading causes of death, hospitalization and disability across the world. Detailed work on child injury in low-income and middle-income countries began more recently and is now indicating priorities for prevention. This study aims to draw attention to the subject with the assessment of the injuries sustained by the study group.

***Methodology: ***Data of the descriptive study was collected at the Emergency Department’s trauma section of a Training and Research Hospital, located in Ankara during the period of October-November 2010. Children visiting the hospital due to an injury under the age of 18 were included to the study group by assent of the child and consent of the parents.

***Results: ***Of the study group 75.3% were boys, 35.31% were 11-15 year of age. The most frequent cause of the injuries were falls among boys (48.3%), girls (50.0%) and, 11-15 years of age (32.8%). Most injured organs (63.6%) were extremities and most frequent damage caused by the injury was fracture (29.5%). Almost half of the injuries (44.0%) occurred in and around the school.

***Conclusion: ***Development and implementation of systematic surveillance is necessary to identify the epidemiologic characteristics of childhood injuries at national level. Definition of the risk factors and protective factors is a priority of countries to prevent such injuries.

## INTRODUCTION

Risk factors and protective factors for individual types of child injury have been identified in high-income countries. The characteristics of children susceptible to injury vary greatly by age, gender, race and socioeconomic status.^1^ With improvements in other areas of child health and better methods of collecting data, it is now clear that injury is a leading cause of child death and ill-health in low-income and middle-income countries (LMIC). The full extent of the problem of injuries in many countries, though, is still not fully understood.^1^

The global childhood unintentional injury surveillance (GCUIS) study was initiated in January 2007. The objective of the study was to determine the frequency and nature of childhood injuries in selected urban settings within LMIC by means of a pilot injury surveillance system.^2^

The study presented in this paper, attempts to enhance the evidence base for describing unintentional child injuries in Ankara, the capital of Turkey.

## METHODOLOGY

During the data collection period (5^th^ October-3^rd^ December 2010), researchers administered the questionnaire at the Emergency Department’s (ED) trauma section of the Training and Research Hospital on weekdays between 08:30 am and 05:30 pm. The Hospital is a big center, located in an easily accessible district of Ankara and has a workload that may reflect the hospitals of the City.^3^

Children under the age of 18 years visiting the hospital due to an injury who (both themselves and their parents) gave oral consent were included to the study group (275 of 290). On the other hand when the child arrived in the hospital alone, only his/her consent was taken (6.5% of the group, n=18, Table-II). Children injured by others intentionally (e.g., stabbings, gunshot wounds, physical violence and sexual abuse), self-injuries or injuries related with the use of substance-alcohol, were excluded from the study. One child died at the trauma section of ED was excluded because the injury was classified as intentional afterwards. 

The questionnaire contained 39 questions, had been prepared by the researchers with the use of national and international sources, including GCUIS form. Data was collected with face to face interview technique. Information was obtained by the interviews with mothers (49.8%) and, fathers (23.6%) mainly. Only 14.2% of the group provided self-reported information. 

Official approval was taken from the Ankara Provincial Health Directorate and the Ethical Board of the Hospital.

## RESULTS

**Fig.1:**
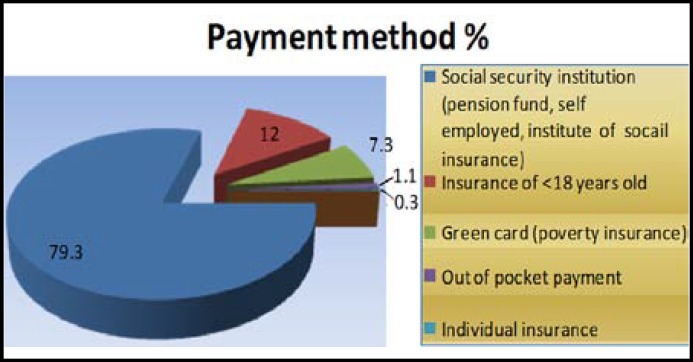
Primary method of payment for care received (n=275).

***Socio demographic characteristics and chronic conditions: ***Boys formed the 75.3% of the study group and, 35.3% was 11-15-year-olds (Table-I). More than half (56.0%) of the children were enrolled to primary school. Of the fathers’ 45.3% and mothers’ 54.9% had 6 years or less schooling. While 83.6% of fathers had a job, 10.2% was unemployed and, most of the mothers were unpaid housewives (88.7%). Two fathers and one mother were dead. Of the parents 94.5% was living together. One mother and five fathers were handicapped (at total 2.2% of the parents). Of the children injured, 5.1% reported a physical handicap or a chronic condition and, 15.3% reported one or more injuries in the last year. Primary method of payment for care received is presented in Fig.1.

***Characteristics and outcomes of injuries: ***More than half of the injuries (58.5%) took place during afternoon, 31.6% between 06:00am-11:59, and most frequent accompanying person during injury was reported as friends (45.8%). Main injury types were fall (48.8%), mainly fall from 50-99 cm height (32.0%) and, shock/compression/sprain (35.6%) both among boys and girls (Table-II). Most frequent damages caused by the injury were fractures (29.5%) and cut /open wounds (17.8%), whereas 18.9% of the injuries did not cause any damage (Table-I). Injured organ was extremity in more than half of the cases (63.6%). 

**Fig.2:**
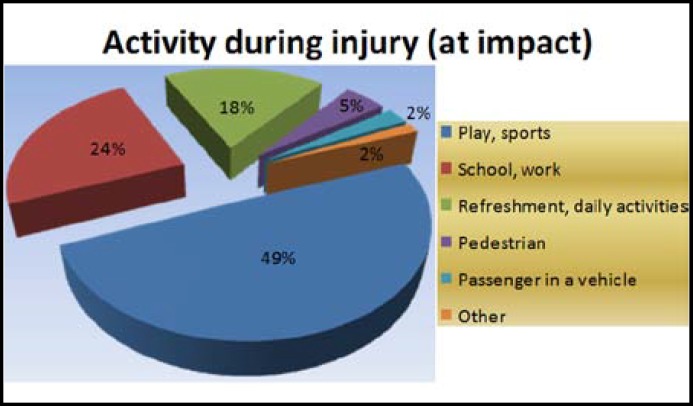
Activity during injury (n=275).

Of the injured children, 42.9% were transported to the hospital in a passenger car and 35.6% were brought to the hospital by their mothers (Table-II). It has been reported that almost half of the children (49%) were playing or engaged in sports during the injury and 44% of the injuries occurred at school (Fig.2, Table-II).

Among study group, falls were most common cause of injury (48.8%) (Table-II, Table-III). Most falls took place at school or at home (41.0%, 29.9% in order) (Table-II). At home, halls or living rooms (37.5%) were most reported places of impact. The most severe fall-related injuries were fractures (42.5%) and cuts, open wounds (14.2%) (Table-III).

Projected effect of injury was short-term temporary disability (=/<6 weeks) in 44.7% of the cases, however there had been one respiratory arrest related death due to fall (Table-III). About one third of the children were treated and discharged (29.1%) and treatment of 69.4% was in progress, 3 cases (out of vehicle traffic accident) needed emergency surgery (Table-III).

Of the 19 children (6.9% of the study group) involved in road traffic injuries, 63.1% (12) were pedestrians and were walking or running during the impact. Two third of the 15 striking vehicles or objects were cars. All the children who suffered from vehicle road traffic injury reported to be seated at the back seat and had not been wearing seat belts. In injuries that occurred while driving a bicycle or motorcycle, nobody was wearing helmets or knee pads. 

Of the study group, 33.1% reported that the child or the family had some first-aid information before the impact. Around one third of the children injured (31.3%) received first-aid, in the 15.9% of the cases first-aid **was** provided by mothers and the most frequent first-aid intervention reported was ice application (12.2%) to the injury location.

## DISCUSSION

Findings of the study are in accordance with the patterns of the detailed work in other regions and countries of the World. Death is the most notable measure of injury but it is neither the only outcome nor the most common. Injury is often graphically represented as a pyramid, with the smallest group, that of death, at the top, hospitalized injury in the middle and the largest group, non-hospitalized injury, at the base.^1^^,^^2^

**Table-I:** Damaged caused by gender and age groups

*Characteristics*	*Fracture*		*Dislocation* */ sprain*		*Cut* */ open* *wound*		*Soft* *tissue injury*		*Burn*		*Internal organ injury*		*Normal* *vital* *signs*		*Other*		*Total*	
	*n*	*%*	*n*	*%*	*n*	*%*	*n*	*%*	*n*	*%*	*n*	*%*	*n*	*%*	*n*	*%*	*n*	*%*
Gender																		
Boy	60	28.9	29	14.0	43	20.8	34	16.4	1	0.5	1	0.5	35	17.0	4	1.9	207	75.3
Girl	21	31.0	15	22.0	6	8.8	6	8.8	0	0.00	0	0.00	17	25.0	3	4.4	68	24.7
Age Groups																		
<1	4	16.0	4	16.0	3	12.0	4	16.0	0	0.0	0	0.0	8	32.0	2	8.0	25	9.0
1-5	13	30.2	7	16.27	9	20.93	2	4.65	1	2.32	0	0.0	9	20.93	2	4.65	43	15.7
6-10	32	36.4	8	9.1	21	23.9	15	17.0	0	0.0	0	0.0	10	11.3	2	2.3	88	32.0
11-15	27	27.8	22	22.7	11	11.3	16	16.5	0	0.0	1	1.0	20	20.7	0	0.0	97	35.3
=>16	5	22.7	3	13.7	5	22.7	3	13.7	0	0.0	0	0.0	5	22.7	1	4.5	22	8.0
Total	81	29.5	44	16.0	49	17.8	40	14.5	1	0.4	1	0.4	52	18.9	7	2.5	275	100.0

**Table-II:** Descriptive data surrounding unintentional injuries of the study group

*Characteristics*	*Fall*		*Shock* * / * *compression* * / sprain*		*Foreign object* */ piercing/* *cutting tool*		*Foreign* *object* *fall*		*Out of vehicle traffic accident*		*In vehicle traffic accident*		*Other*		*Total*	
	*n*	*%*	*n*	*%*	*n*	*%*	*n*	*%*	*n*	*%*	*n*	*%*	*n*	*%*	*n*	*%*
Mode of transit to hospital																
Passenger car	55	41.0	46	46.9	10	62.6	1	20.0	2	15.4	3	50.0	1	33.3	118	42.9
Public transportation	31	23.1	36	36.7	4	25.0	0	0	0	0	0	0	1	33.3	72	26.1
Ambulance	23	17.2	4	4.1	1	5.7	3	60.0	9	69.2	2	33.3	0	0	42	15.3
Taxi	16	12,0	3	3.1	0	0	1	20.0	2	15.4	1	16.7	1	33.3	24	8.8
Pedestrian	9	6.7	9	9.2	1	5.7	0	0	0	0	0	0	0	0	19	6.9
Person took child to emergency department																
Mother,	48	35.8	37	37.8	6	37.5	3	60.0	2	15.4	1	16.7	1	33.3	98	35.6
Father,	20	14.9	17	17.3	2	12.5	1	20.0	1	7.7	0	0	0	0	41	14.9
Other family members/ Parents	52	38.8	31	31.6	5	31.3	1	20.0	3	23.0	4	66.6	2	66.7	98	35.6
Friend/teacher	8	6.0	9	9.2	2	12.5	0	0	0	0	1	16.7	0	0	20	7.4
Other	6	4.5	4	4.1	1	6.2	0	0	7	53.9	0	0	0	0	18	6.5
Place injury occurred																
School	55	41.0	59	60.2	6	37.5	0	0	0	0	0	0	0	0	120	43.6
Home	40	29.9	22	22.5	4	25.0	4	80.0	0	0	0	0	3	100	73	26.5
Road/ street	21	15.7	6	6.1	6	37.5	1	20.0	13	100	6	100	0	0	53	19.3
Sports/ play area	12	8.9	10	10.2	0	0	0	0	0	0	0	0	0	0	22	8.0
Industrial/public building	6	4.5	1	1.0	0	0	0	0	0	0	0	0	0	0	7	2.6
Total	134	48.8	98	35.6	16	5.8	5	1.8	13	4.7	6	2.1	3	1.2	275	100.0

**Table-III:** Characteristics of unintentional injuries of the study group

*Characteristics*	*Fall*		*Shock* * / * *compression* * / sprain*		*Foreign object * */ piercing / cutting tool*		*Foreign* *object* *fall*		*Out of vehicle traffic accident*		*In vehicle traffic accident*		*Other*		*Total*	
	*n*	*%*	*n*	*%*	*n*	*%*	*n*	*%*	*n*	*%*	*n*	*%*	*n*	*%*	*n*	*%*
Damage caused by the injury																
Fracture	57	42.5	13	13.3	0	0	2	40.0	7	53.8	2	33.3	0	0	81	29.5
Dislocation/ sprain	15	11.2	29	29.6	0	0	0	0	0	0	0	0	0	0	44	16.0
Cut/open wound	19	14.2	10	10.2	16	100.0	0	0	2	15.4	2	33.3	0	0	49	17.8
Soft tissue injury	15	11.2	19	19.4	0	0	2	40.0	2	15.4	2	33.3	0	0	40	14.5
Burn	0	0	0	0	0	0	0	0	0	0	0	0	1	33.3	1	0.4
Internal organ injury	1	0.75	0	0	0	0	0	0	0	0	0	0	0	0	1	0.4
No Damage	22	16.4	26	26.5	0	0	1	20.0	2	15.4	0	0	1	33.3	52	18.9
Others*	5	3.7	1	1.0	0	0	0	0	0	0	0	0	1	33.3	7	2.5
Outcome of injury																
Treated and discharged home	38	28.4	33	33.7	5	31.25	0	0	3	23.1	1	16.7	0	0	80	29.1
Treatment in progress	95	70.9	65	66.3	11	68.75	5	100	7	53.8	5	83.3	3	100	191	69.4
Emergency surgery	0	0	0	0	0	0	0	0	3	23.1	0	0	0	0	3	1.1
Died in ED	1	0.7	0	0	0	0	0	0	0	0	0	0	0	0	1	0.4
Projected effect of injury																
Normal vital signs	13	9.7	13	13.3	0	0	0	0	1	7.7	0	0	0	0	27	9.8
No significant disability	41	30.6	55	56.1	10	62.5	0	0	1	7.7	0	0	1	33.3	108	39.3
Short-term temporary disability	71	53.0	30	30.6	6	37.5	5	100	4	30.8	5	83.3	2	66.7	123	44.7
Long-term temporary disability	8	6.0	0	0	0	0	0	0	7	53.8	1	16.7	0	0	16	5.8
Death	1	0.7	0	0	0	0	0	0	0	0	0	0	0	0	1	0.4
Total	134	48.8	98	35.6	16	5.8	5	1.8	13	4.7	6	2.1	3	1.2	275	100.0

ED, emergency department;

Short-term temporary disability, (6 =< weeks);

Long-term temporary disability, (> 6 weeks).

* Others include (1respiratory arrest due to fall, 1 temporary hearing loss due to shock, mechanical forces).

UNICEF and the Alliance for Safe Children have examined health histories for two and a quarter million people in five countries of South and East Asia. The combined data show that, for children under 18 years of age, for each death there are 12 children admitted to hospital or permanently disabled and 34 children who needed medical care or missed school or work because of an injury.^1^^,^^2^^,^^4^

Data from developed countries indicate that, from birth onwards, males have higher rates of injury than females, for all types of injury.^1^ Also according to the Childhood Injury Report: 2000-2006, among 0-19 year olds, nonfatal injury rates were higher among males than females, while the rates were approximately the same for those less than one year in the United States.^5^

Globally, 50% of the total number of DALYs lost due to falls occur in children less than 15 years of age. However, the burden of childhood falls is largely explained by the morbidity and disabilities that may persist for life.^1^ In the United States each year, approximately 2.8 million children had an initial emergency department visit for injuries from a fall. For children less than one year of age, falls accounted for over 50% of nonfatal injuries.^5^

Most of the (44%) injuries occurred at school in our study. Findings of Health Behavior in School-aged children in Turkey Study (2006) indicate that children are injured most frequently at school, at sport clubs or at homes.^6^

## CONCLUSION

Childhood injuries are predicted by a set of interrelated sociodemographic, cognitive, behavioral, and child-related factors.^2^ Hospitals in low-income countries bear a substantial burden of childhood injuries, and systematic surveillance is required to identify the epidemiological distribution of such injuries and understand their risk factors.^2^

Deficiencies in four areas have stymied injury prevention efforts of the past and continue to be a problem today in HIC, are also important in LMIC: (1) the absence of detailed and reliable data on nonfatal injuries; (2) the lack of training to prepare a multidisciplinary group of professionals to enter the field of injury prevention; (3) inadequate funding; and (4) the lack of coordinated prevention efforts. Development and expansion of efforts in these areas will be critical in reducing or further reducing the occurrence of childhood injuries in the coming years.^7^ Besides educational, structural improvements and enforcement of legislation and regulation^8^^-^^10^, building up a national surveillance system seems crucial to reduce the prevalence of unintentional childhood injuries, a serious public health issue.

***Abbreviations:***

EDs: Emergency Departments.

GCUIS: Global Childhood Unintentional Injury Surveillance.

HIC: High-Income Countries.

LMIC: Low- and Middle-Income Countries.

SSI: Social Security Institution.

***Limitations: ***Findings are limited with the reporting’s of the patients, data collection period and the schedule. No funding support was received during the study.

## Authors Contributions

All authors contributed equally to the paper. Piyal and Yilmaz designed the study, Piyal, Akdur and Ocaktan wrote the paper. Yilmaz, Ikinci and Celik collected and assessed the data and prepared the tables and graphs.

## Notes

The manuscript has not been published or submitted elsewhere. Some of the descriptive findings of the study were shared with a poster presentation on the 14th National Public Health Congress (4-7 October 2011).
